# Integrative analysis of neuroblastoma and pheochromocytoma genomics data

**DOI:** 10.1186/1755-8794-5-48

**Published:** 2012-10-29

**Authors:** Peter M Szabó, Miklós Pintér, Diana Rita Szabó, Adrienn Zsippai, Attila Patócs, András Falus, Károly Rácz, Peter Igaz

**Affiliations:** 1 2nd Department of Medicine, Faculty of Medicine, Semmelweis University, Szentkirályi str. 46, Budapest, H-1088, Hungary; 2 Department of Mathematics, Corvinus University of Budapest, Fővám sqr. 8, Budapest, H-1093, Hungary; 3 Molecular Medicine Research Group, Hungarian Academy of Sciences, Szentkirályi str. 46, Budapest, H-1088, Hungary; 4 Department of Genetics, Cell- and Immunobiology, Semmelweis University, Nagyvárad sqr. 4, Budapest, H-1089, Hungary

**Keywords:** Pheochromocytoma, Neuroblastoma, Functional genomics, microRNA, Cooperative game theory

## Abstract

**Background:**

Pheochromocytoma and neuroblastoma are the most common neural crest-derived tumors in adults and children, respectively. We have performed a large-scale in silico analysis of altogether 1784 neuroblastoma and 531 pheochromocytoma samples to establish similarities and differences using analysis of mRNA and microRNA expression, chromosome aberrations and a novel bioinformatics analysis based on cooperative game theory.

**Methods:**

Datasets obtained from Gene Expression Omnibus and ArrayExpress have been subjected to a complex bioinformatics analysis using GeneSpring, Gene Set Enrichment Analysis, Ingenuity Pathway Analysis and own software.

**Results:**

Comparison of neuroblastoma and pheochromocytoma with other tumors revealed the overexpression of genes involved in development of noradrenergic cells. Among these, the significance of paired-like homeobox 2b in pheochromocytoma has not been reported previously. The analysis of similar expression patterns in neuroblastoma and pheochromocytoma revealed the same anti-apoptotic strategies in these tumors. Cancer regulation by stathmin turned out to be the major difference between pheochromocytoma and neuroblastoma. Underexpression of genes involved in neuronal cell-cell interactions was observed in unfavorable neuroblastoma. By the comparison of hypoxia- and Ras-associated pheochromocytoma, we have found that enhanced insulin like growth factor 1 signaling may be responsible for the activation of Src homology 2 domain containing transforming protein 1, the main co-factor of RET. Hypoxia induced factor 1α and vascular endothelial growth factor signaling included the most prominent gene expression changes between von Hippel-Lindau- and multiple endocrine neoplasia type 2A-associated pheochromocytoma.

**Conclusions:**

These pathways include previously undescribed pathomechanisms of neuroblastoma and pheochromocytoma and associated gene products may serve as diagnostic markers and therapeutic targets.

## Background

Neuroblastoma (NB) and pheochromocytoma (PCC) are neural crest-derived tumors that are both associated with significant morbidity and mortality.

NB is the most frequent malignant tumor in children accounting for 15% of childhood cancer mortality. Approximately 38% of primary tumors are localized in the adrenal medulla and 1-2% of newly diagnosed NB cases are related to familial history of disease [1]. The genetic aberration most consistently associated with poor outcome in NB is the amplification of v-myc myelocytomatosis viral related oncogene (*MYCN*), which occurs in 20% of primary tumors. Chromosomal aberrations as deletion of chromosomes 1p, 11q and gain of 17q are also associated with poor prognosis. The International Neuroblastoma Staging System is most commonly used for NB staging. Stages 1 and 2 represent localized primary tumors, whereas the primary tumor is unresectable in stage 3 with or without lymph node infiltration, and stage 4 represents any primary tumor with dissemination to distant lymph nodes, bone marrow, liver, skin or other organs. The special 4S phenotype is characterized by localized tumor in infants younger than 1 year, with dissemination limited to skin, liver, or bone marrow, and mostly spontaneous regression [1].

PCC is a rare tumor, mostly occurring in adults and in the adrenals. Approximately 10% of PCC are malignant. PCC mostly develops in the adrenal. Extraadrenal PCC is termed paraganglioma (PGL). 25–30% of all cases are related to monogenic hereditary tumor syndromes including multiple endocrine neoplasia 2 (MEN2), von Hippel-Lindau syndrome (VHL), neurofibromatosis type 1 (NF1) and the group of PGL syndromes caused by mutations in genes encoding succinate dehydrogenase (SDH) subunits and associated factors [2,3]. Novel genes like *TMEM127* and *MAX* have also been described [4,5]. Two major pathogenic pathways have been established in these syndromes: hypoxia pathway (VHL and PGL syndromes) and the pathway involving Ras and mechanistic target of rapamycin (*mTOR*) activation (MEN2 and NF1). These pathways seem to be implicated in the pathogenesis of sporadic PCC, as well [5,6].

Despite many data on these tumors, we are still far from an overall picture on tumorigenesis. Several studies applying functional genomics approaches have been performed on these tumors to date including the analysis of mRNA and microRNA expression patterns, and studies on chromosome aberrations. In silico analysis of functional genomics studies might be a useful way to decipher common pathogenic pathways and differences [7].

In this study, we have performed a large scale in silico analysis of functional genomics data including a large number of NB and PCC samples to establish their similarities, differences, and to compare their different subgroups using analysis of gene expression, microRNA expression and their potential mRNA targets, and chromosome aberrations. For this purpose, we have applied GeneSpring, Gene Set Enrichment Analysis (GSEA), and Ingenuity Pathway Analysis (IPA) software. We have also applied a novel bioinformatical analysis based on cooperative game theory for the establishment of relevant gene expression changes. In this approach developed by Moretti et al. [8], genes serve as players and microarray measurements are called cooperative games. By the cooperative game theory analysis we are able to identify the power or relevance index of each gene in inducing the investigated pathological state.

## Methods

### Data sets

Microarray data sets were obtained from publicly available databases (Gene Expression Omnibus (GEO, http://www.ncbi.nlm.nih.gov/gds) and ArrayExpress (http://www.ebi.ac.uk/arrayexpress)). Data on NB were collected from eight studies including 273 samples on three different platforms [9-16]. These were grouped by stages and and *MYCN* status (Table 1). Data on PCC were collected from three studies [5,6,17] including 330 samples analyzed on four different platforms (Table 2). Samples were grouped by the mutations. In one case [6], the sample set contained malignant PCC, however, due to the incomplete sample annotation we were unable to identify these. In another case [17], only head-neck PGL samples were investigated that were compared to NB samples. Duplicate samples were removed from the analysis in both NB and PCC cases. For the taxonomical classification of NB and PCC, we have analyzed 368 random selected samples from 54 different types of endo-, meso- and ectodermic tumors and normal tissues from 17 studies [11,12,18-30] and 39 NB and 75 PCC samples measured on the same Affymetrix HG-U133A Array (Table 3).

**Table 1:** Characteristics of the analyzed NB microarray data sets

**Study**	**Accesion No.**	**Platform**	**Samples**	**Stages**	***MYCN***
Łastowska et al., 2007 [15]	GEO: GSE13136	Affymetrix HG-U133 Plus 2.0 Array	30 neuroblastoma	Stage 1: 4, Stage 2: 2, Stage 3: 1, Stage 4: 20, Stage 4S: 3	*MYCN*-amplified: 10, MYC-non-amplified: 20
Hiyama et al., 2004 [13]	GEO: GSE16237		51 neuroblastoma	Stage 1: 21, Stage 2: 6, Stage 3: 5, Stage 4: 13, Stage 4S: 6	*MYCN*-amplified: 7, MYC-non-amplified: 44
Janoueix-Lerosey et al., 2008 [14]	GEO: GSE12460		52 neuroblastoma, 8 ganglioneuroblastoma, 3 ganglioneuroma, 1 unknown	Stage 1: 10, Stage 2: 10, Stage 3: 7, Stage 4: 16, Stage 4S: 8, unknown: 1	*MYCN*-amplified: 14, MYC-non-amplified: 32, unknown: 6
Wilzén et al., 2009 [10]	ArrayExpress: E-MEXP-2250	Affymetrix HG-U133A Array	6 neuroblastoma	Stage 1: 1, Stage 2: 1, Stage 3: 4	unknown: 6
De Preter et al., 2006 [12]	ArrayExpress: E-MEXP-669		18 neuroblastoma, 3 adrenal cortex, 3 adrenal neuroblast	Stage 1: 4, Stage 3: 3, Stage 4: 10, Stage 4S: 1	*MYCN*-amplified: 5, *MYCN*-non-amplified: 13
Albino et al., 2008 [11]	GEO: GSE7529		10 neuroblastoma, 3 ganglioneuroblastoma, 6 ganglioneuroma, 9 microdissected tumors	unknown: 10	unknown: 10
Staege et al., 2004 [16]	GEO: GSE1825		5 neuroblastoma, 5 Ewing family tumor	Stage 1: 3, Stage 3: 1, Stage 4: 1	unknown: 5
Wang et al., 2006 [9]	GEO: GSE1825	Affymetrix HG-U95 Version 2 Array	101 neuroblastoma, 1 fetal brain	Stage 1: 28, Stage 3: 23, Stage 4: 50	*MYCN*-amplified: 20, *MYCN*-non-amplified: 81

**Table 2:** Characteristics of the analyzed PCC microarray data sets

**Study**	**Accesion No.**	**Platform**	**Samples**	**Mutation**
Burnichon et al., 2011 [6]	ArrayExpress: E-MTAB-733	Affymetrix HG-U133 Plus 2.0 Array	188 pheochromocytoma	Sporadic: 95, MEN2A: 9, SDHA: 1, SDHB: 17, SDHC: 2, SDHD: 3, VHL: 27, NF1: 9, Other: 25
Qin et al., 2010 [5]	GEO: GSE19987	Affymetrix HG-U133A Array	75 pheochromocytoma	Sporadic: 35, MEN2A: 9, SDHB: 10, SDHD: 2, VHL: 6, NF1: 2, Other: 11
Qin et al., 2010 [5]	GEO: GSE19987	Affymetrix HG-U133A 2.0 Array	50 pheochromocytoma	Sporadic: 36, MEN2A: 3, MEN2B:1, SDHB: 4, VHL: 3, NF1: 2, Other: 1
Hensen et al., 2009 [17]	GEO: GSE12921	Affymetrix HG-U95 Version 2 Array	17 pheochromocytoma	Sporadic: 7, SDHD: 6, PGL2: 4

**Table 3:** Characteristics of the datasets used for cluster analysis

**Study**	**Accesion No.**	**Samples**
Henderson et al., 2005 [23]	ArrayExpress: E-MEXP-353	4 alveolar rhabdomyosarcoma, 5 chondroblastoma, 4 chondromyxoid fibroma, 7 chondrosarcoma, 4 chondroma, 3 dedifferentiated chondrosarcoma, 3 embryonal rhabdomyosarcoma, 5 Ewing tumor, 5 fibromatosis, 8 leiomyosarcoma, 3 lipoma, 4 malignant peripheral nerve sheath tumor, 10 monophasic synovial sarcoma, 7 myxoid liposarcoma, 4 neurofibroma, 11 osteosarcoma, 3 sarcoma, 4 schwannoma, 3 well-differentiated liposarcoma
Giordano et al., 2006 [21]	ArrayExpress: E-GEOD-27155	4 anaplastic thyroid carcinoma, 10 follicular thyroid adenoma, 2 follicular thyroid carcinoma, 2 medullary thyroid carcinoma, 4 normal thyroid, 7 oncocytic thyroid adenoma, 8 oncocytic thyroid carcinoma, 13 papillary thyroid carcinoma
Freije et al., 2004 [20]	GEO: GSE4412	6 astrocytoma, 10 glioblastoma, 5 anaplastic mixed oligo-astrocytoma, 10 anaplastic oligodendroglioma
Tirode et al., 2007 [29]	ArrayExpress: E-GEOD-7007	11 Ewing tumor
Jones et al., 2005 [24]	ArrayExpress: E-GEOD-15641	6 chromophobe RCC, 10 clear cell RCC, 10 oncocytoma, 10 papillary RCC, 8 transitional cell carcinoma
Lenburg et al., 2003 [25]	ArrayExpress: E-GEOD-781	16 clear cell RCC, 1 normal kidney
De Preter et al., 2006 [12]	ArrayExpress: E-MEXP-669	3 adrenal cortex, 3 neuroblast
Albino et al., 2008 [11]	GEO: GSE7529	3 ganglioneuroblastoma, 6 ganglioneuroma
Boyault et al., 2007 [18]	ArrayExpress: E-TABM-36	17 hepatocellular carcinoma, 3 hepatocellular adenoma
Parent et al., 2008 [27]	ArrayÍExpress: E-GEOD-7142	4 hypothalamic hamartoma
Wong et al., 2005 [30]	ArrayExpress: E-GEOD-12907	5 juvenile pilocytic astrocytoma, 3 normal cerebellar tissue, 2 normal fetal brain
Su et al., 2007 [28]	ArrayExpress: E-GEOD-7670	1 large cell lung carcinoma, 14 lung adenocarcinoma, 12 normal lung
Monzon et al., 2009 [26]	GEO: GSE12630	1 medullary thyroid carcinoma
Harlin et al., 2009 [22]	ArrayExpress: E-GEOD-12627	13 melanoma
Salomon et al.,*	ArrayExpress: E-GEOD-4780	5 meningioma
Corbin et al., 2009 [19]	ArrayExpress: E-TABM-53	9 normal kidney, 11 Wilms' tumor
Mangiola et al.,*	ArrayExpress: E-GEOD-13276	3 normal white matter

Asterisks mark studies where references were unavailable.

Further significant gene sets for NB and PCC have been retrieved from studies [31-45], where raw genomic data were unavailable. These studies (10 for NB and 5 for PCC) have been identified by literature search (http://www.ncbi.nlm.nih.gov/pubmed) and included microarray-based gene expression data from 1511 NB and 201 PCC samples. The list of significant genes was unavailable in the study by Brown et al. [37], and we have therefore calculated it from normalized data by applying SAM (Table 4).

**Table 4:** Characteristics of gene sets in publications where whole microarray data were unavailable

**Study**	**Number and distribution of samples**	**Gene list**
Asgharzadeh et al., 2006 [35]	102 NB	55 gene model for the prediction of high molecular risk NB
Berwanger et al., 2002 [36]	Stage 1: 19, stage 4: 21 NB	36 genes differently expressed between stage 1 and stage 4 NB
Fischer et al., 2010 [40]	81 unfavorable, 8 favorable NB, without 11q aberration	163 genes differently expressed between favorable and unfavorable NB
Oberthuer et al., 2006 [42]	251 NB, among these 77 was used to construction of a gene expression–based classifier and 177 to validate it	60 genes with higher than 2-fold expression changes from 144 gene predictor of favorable and unfavorable NB
Oberthuer et al., 2007 [33]	79 favorable and 48 unfavorable NB	5 genes with higher than 2-fold expression changes from 349 gene predictor of favorable and unfavorable NB
Ohira et al., 2005 [34]	136 NB	41 top-ranked genes used for prediction of 2 year and 5 year prognosis of NB
Schramm et al., 2005 [31]	59 *MYCN*-non-amplified, 9 *MYCN*-amplified NB	28 top-ranked genes to discriminate *MYCN*-non-amplified and *MYCN*-amplified NB
	Stage 1: 20, stage 2: 16, stage 3:7, stage 4: 15, stage 4S: 10 NB	30 top-ranked genes to discriminate stage 1–2 and stage 3–4 NB
		22 top-ranked genes to discriminate stage 4S and stage 4 NB
Takita et al., 2004 [43]	stage 1: 9, stage 2: 4, stage 4: 6, stage 4S: 1 NB	60 top-ranked genes to discriminate stage 1–2 and stage 4 NB
Thorell et al., 2009 [44]	16 favorable and 15 unfavorable NB	81 genes differently expressed between favorable and unfavorable NB, validated by QRT-PCR
Vermeulen et al., 2009 [45]	579 NB	59 prognostic genes for high risk NB
Brown et al., 2008 [37]	12 MEN2A, 11 VHL PCC	17 genes differently expressed between MEN2A and VHL by SAM (fold change: 2-fold, delta lower than 0.05)
Eisenhofer et al., 2004 [38]	7 MEN2A, 12 VHL PCC	493 genes differently expressed between MEN2A and VHL PCC
Eisenhofer et al., 2008 [39]	32 MEN2A, 47 VHL PCC	42 selected pathway components differently expressed between MEN2A and VHL PCC
Huynh et al., 2006 [41]	13 MEN2A, 18 VHL PCC	15 genes selected differently expressed genes between MEN2A and VHL PCC
López-Jiménez et al., 2010 [32]	8 SDHB, 1 SDHC, 4 SDHD, 15 VHL, 17 RET, 4 NF1	444 genes differently expressed between VHL/SDH and RET/NF1 PCC

Analysis of raw microRNA expression data was possible in both NB and PCC. MicroRNA data for NB were obtained from the study of Schulte et al. [46] including intensity values of 307 microRNAs in 69 samples. To identify differences in microRNA expression between these groups, one-way ANOVA with Scheffé’s post hoc test was used. Statistical analysis was performed by Statistica 8 software (StatSoft Inc., Tulsa, OK, USA). For the analysis of microRNA targets in PCC, we have used our previously published data [47].

Altogether we have analyzed gene expression data from 1784 NB and 531 PCC tissues in our in silico analysis.

### Statistical analysis of mRNA profiling studies

For the classification of tumors, hierarchical clustering was performed on the average of samples within the groups by calculating Euclidean, Squared Euclidean, Manhattan, Differential, Chebyshev distance metric and using the averaged linkage rule. Cluster analysis and all subsequent statistical analyses were performed by Genespring GX 10.5 software (Agilent Tech Inc., Santa Clara, CA, USA).

Identification of gene sets differentially expressed between NB, PCC and other tissues, different stages of NB and different PCC subtypes was carried out by one-way ANOVA. Each ANOVA was followed by Tukey’s post hoc test for all pairwise multiple comparisons. Unpaired *t*-test was used for the comparison of *MYCN*-amplifying, non-amplifying and VHL/SDH-, MEN2/NF1- related PCC. Fold change filter was set to 2-fold in each comparison. Furthermore, Benjamini-Hochberg multiple testing correction (corrected p<0.05) was performed to minimize false positive cases.

### Search for similarities between NB and PCC mRNA expression patterns

To identify genes with highly similar expression levels between NB and PCC samples, we have used the criteria established by Cheng et al. [48] for the identification of reference genes: i., raw intensity value is higher than 20 percent in at least 80% of samples in each group. ii., coefficient of variation is lower than 0.3, and iii. fold change is lower than 1.2. Genes previously identified by Cheng et al. [48] as reference (housekeeping) genes in 4804 samples from 13 different tissues including normal and tumor samples have been filtered out from gene lists. The remaining genes were loaded into Ingenuity Pathway Analysis (IPA). The reference gene selection was performed by an own software written in Java program language.

### Cooperative game theory-based analysis of microarray studies

The main goal of cooperative game theory analysis on microarray samples is the investigation of gene involvement in disease pathogenesis based on a mathematical model. In this analysis, it is initially hypothesized that all genes with more than two-fold expression changes between an investigated sample and the reference sample set play equal role in the pathogenesis, as their cooperation results in a different status. Next, the most frequently involved (playing) genes are searched for. By this approach we are able to identify the most relevant genes (players) in the pathogenesis, because the calculated subsets of genes usually act (change their expression) cooperatively [8].

To calculate the relevance index of genes we have used the same procedure as Albino et al. [11]. We have generated Boolean matrices where the columns represented the samples (games) and the rows represented the genes (players). The values of the matrices were set to 1 if the expression of the gene in the given sample was higher than 2-fold compared to the other group and 0 if not. Shapley values [49] for each gene were calculated as we divided each value in the columns of the Boolean matrices by the sum of the given column and averaged these values by the rows. Finally, each Shapley value was divided by the highest one. We performed these calculations vice versa between the compared groups and genes with values higher than 0.6 in both calculations were selected for further analysis. Gene sets generated by this approach were loaded into IPA. Calculations were performed by an own program written in Java program language.

### Gene set enrichment analysis (GSEA)

GSEA analyzes gene expression data and determines whether a particular set of genes is over- or underrepresented in the samples compared [50]. GSEA was performed by GSEA software v2.0 (http://www.broad.mit.edu) in pairwise comparisons of NB versus PCC, different stages of NB, *MYCN*-non-amplifying vs. *MYCN*-amplifying NB, SDH/VHL vs. MEN2/NF1 PCC and MEN2A vs. VHL-associated PCC. Gene expression results derived from microarray experiments were correlated with chromosomal data and microRNA gene sets. GSEA was performed using gene set permutation type as default and the number of permutation was set to 1000. Statistical significance levels were defined as nominal p-value 0.05 and false discovery rate 0.25.

### Correlation of GSEA results retrieved from publicly available microarray data with cytogenetic changes

To correlate gene expression changes to chromosomal aberrations we have compared significantly enriched chromosomal gene sets by GSEA with the list of previously published chromosomal aberrations for the given tumor types. From the gene lists of these overlapping chromosomal regions, we have selected the most prominent expression changes by Leading Edge Analysis (LEA) using GSEA software v2.0 (http://www.broad.mit.edu). The leading edge subset of genes can be defined as the core accounting for the enrichment signal of the significant chromosomal gene set. This method has been described in our previous study [7] and defined strict cut-off parameters to identify genes harbored on aberrant chromosome regions showing the most prominent expression changes [51].

### Correlation of GSEA results retrieved from publicly available microarray data with microRNA expression

Identification of predicted microRNA targets was performed using TargetScan 5.2 (http://www.targetscan.org), PicTar (http://pictar.org) and MicroCosm Targets (http://www.ebi.ac.uk/enright-srv/microcosm/htdocs/targets/v5/) databases. The outputs from these databases were merged by an own program written in C++ program language.

Further analysis aimed at tissue specific microRNA target prediction has been performed as described in our previous study [52]. Briefly, unexpressed mRNAs (intensity value is lower than 20 percentile in at least 20 percent of the samples in both groups) were filtered out from target lists. For all significantly differentially expressed microRNAs within the two groups, we have generated gene sets from their expressed target genes. Then GSEA was performed and LEA was used to select potential mRNA targets with inverse expression alterations as their regulatory microRNAs. Pairwise comparison was performed between different stages and *MYCN*-non-amplifying and amplifying NB and between SDH/VHL- and MEN2/NF1- and MEN2A-, VHL-related PCC. All analyses were performed by own programs written in Java program language.

### Pathway analysis

We have used Ingenuity Pathway Analysis (IPA) to decipher the possible biological relevance of gene expression changes established. (Ingenuity Systems, http://www.ingenuity.com; Redwood City, CA, USA). Gene sets established both by in silico analysis of mRNA expression (significant expression changes, unchanged expression, results of cooperative game theory analysis and data collected by literature search), GSEA of microRNA and comparative genomic hybridization (CGH) gene sets were subjected to IPA and significant pathways (p<0.05) were compared to each other.

## Results

We have performed several analyses including a taxonomical analysis based on the gene expression profile of NB and PCC samples and other tissues, and tried to characterize the most prominent differences between neural crest-derived tumors and other tissues. We have compared NB and PCC data to establish their differences and similarities, moreover within the NB and PCC groups, data from different NB stages and from PCC subgroups, respectively, have been analyzed (Additional file 1).

### Differences between NB, PCC and other tumors and similarities of NB and PCC tissues

**Figure 1:**
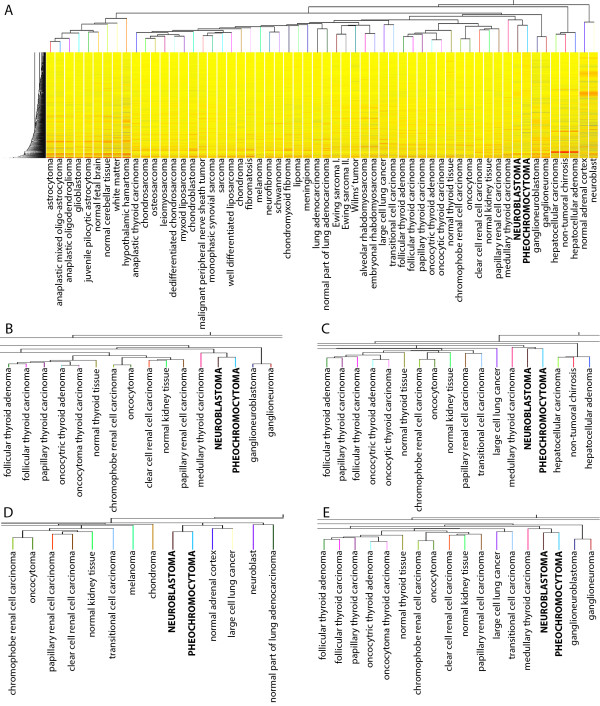
Cluster analysis of NB and PCC with 369 randomly selected samples from tumors and normal tissues representing 54 endo-, meso- and ectodermic tissues, by calculating A: Euclidean, B: Squared Euclidean, C: Manhattan, D: Differential, E: Chebyshev distance metric and using the averaged linkage rule.

To categorize NB and PCC among different endo-, meso-, and ectodermic tumors, unsupervised hierarchical clustering was performed on 54 different groups of normal tissues and tumors. By this approach, NB and PCC were clustered close to each other underlining their similarity in gene expression patterns (Figure 1).

By the comparison of NB or PCC groups with the investigated 54 normal tissues and tumor types, we have identified 36 genes significantly differentially expressed in more than 80% of comparisons (Additional file 2). By the manual inspection of these 36 genes, they could be clearly categorized as genes involved in catecholamine synthesis, transport and storage: dopamine beta-hydroxylase (*DBH*), tyrosine hydroxylase (*TH*), chromogranin A (*CHGA*), chromogranin B (*CHGB*), solute carrier family 6 member 2 (*SLC6A2*), solute carrier family 18 member 1 (*SLC18A1*), and transcription factors and homeobox genes involved in neural crest-derived cell development: paired-like homeobox 2a and 2b (*PHOX2A, PHOX2B*), GATA binding protein 2 and 3 (*GATA2*, *GATA3*), heart and neural crest derivatives expressed 2 (*HAND2*).

To identify genes responsible for the similarities of NB and PCC gene expression patterns, we have searched for genes expressed in both tumors with the lowest fold change and variation. The previously published [48] reference (or housekeeping) genes were filtered out from these gene lists to select genes with unchanged expression specifically between NB and PCC. The analysis was performed on three different platforms. By this approach we have identified 62 genes that were common in at least two comparisons (Additional file 3). By IPA of these gene sets, death receptor signaling was the most significant common pathway in each comparison.

### Differences between NB and PCC tissues

The comparison of NB and PCC was possible on three platforms, and by the comparison of these gene lists we have identified 758 common expression changes in at least two comparisons (Additional file 4), for example: anaplastic lymphoma receptor tyrosine kinase (*ALK*), neurotrophic tyrosine kinase, receptor, type 3 (*NTRK3*), tubulin, beta 2A-C, 3 (*TUBB2A*, *TUBB2B*, *TUBB2C*, *TUBB3*). By IPA, cancer regulation by stathmin1 has been identified as the most significant pathway between PCC and NB.

### Expression changes between different subgroups of NB

The comparison of significant gene lists between stages 1 and 4 was possible on three different platforms. Within these, we have found 28 common expression changes in at least two studies (Additional file 5). The comparison of stage 4S and stage 4 NB was possible on one platform. The list of significantly differentially expressed genes was compared to the gene list reported by Schramm et al. [31] and 5 common genes have been found in both, for example: aldehyde dehydrogenase 3 family member A2 (*ALDH3A2*) and phosphoinositide-3-kinase regulatory subunit 1 (*PIK3R1*) (Additional file 5).

The analysis of gene expression profiles of *MYCN*-non-amplifying and *MYCN*-amplifying NB was possible on three platforms. The comparison of these revealed 259 common significant expression changes in at least two studies. To identify the most reliable expression changes, we intersected this gene set with a further one, reported by Schramm et al. [31]. By this approach, we have identified nine genes with significant expression changes that were common in all four gene sets, for example: DEAD box polypeptide 1 (*DDX1*), *MYCN*, ornithine decarboxylase 1 (*ODC1*), transketolase (*TKT*) (Additional file 5).

By the comparison of gene lists revealed from the statistical analysis of early versus late stage, *MYCN*-non-amplifying versus *MYCN*-amplifying and favorable versus unfavorable NB samples and from the literature search, we have found 519 genes that were reported at least twice in these gene lists (Additional file 5). Among these, the most prevalent genes were: *NTRK1*, *MYCN*, secretogranin II (*SCG2*), pleiotrophin (*PTN*), neuronal cell adhesion molecule (*NRCAM*) and *ODC1*. The IPA of significant gene expression changes, cooperative game theory analysis, chromosomal changes and microRNA affected genes revealed the axonal guidance as the most significant pathway in the pathogenesis of unfavorable NB.

### Significant expression changes between VHL/SDH and MEN2/NF1 PCC

The comparison of VHL/SDH- and MEN2/NF1-associated PCC was possible on three platforms. To generate a more reliable dataset of significant expression changes, we supplemented these gene lists with a further one, found by literature search [32]. By this approach, we have identified 240 common significant gene expression changes in at least two gene lists (Additional file 6), and among these 9 were common in all four comparisons, for example: phenylethanolamine N-methyltransferase (*PNMT*), ret proto-oncogene (*RET*), Src homology 2 domain containing transforming protein 1 (*SHC1*). By IPA, IGF1 (insulin-like growth factor 1) signaling turned out to be the most significantly influenced pathogenic pathway between SDH/VHL and MEN2/NF1 PCC.

### Significant expression changes between MEN2A and VHL PCC

The comparison of MEN2A- and VHL-associated PCC was possible on three platforms, and a further dataset was analyzed by SAM (significance analysis of microarrays), moreover three significant gene lists were collected from literature search. By the comparison of these seven gene lists, we have identified 162 genes which were common in at least two gene lists, for example: *CHGB*, neural cell adhesion molecule 1 (*NCAM1*), placental growth factor (*PGF*), *PNMT*, vascular endothelial growth factor (*VEGF*) (Additional file 7). By the IPA of significant gene expression changes, cooperative game theory analysis, chromosomal aberrations and microRNA affected genes, the most significant pathogenic pathway was VEGF and hypoxia inducible factor 1 alpha (HIF1-α) signaling.

### Cooperative game theory analysis

The number of index genes revealed from cooperative game theory analysis [8] was similar to the number of significant gene expression changes and showed good overlap with them. In average, more than 40% of significant gene expression changes were identified as index genes by cooperative game theory analysis in different comparisons. The pathway analysis of index genes always revealed the same canonical pathways as significant gene lists and supplemented these with several new members. These observations support the applicability of cooperative game theory in the in silico analysis of gene expression.

## Discussion

### Comparison of NB and PCC with other tumors

By the cluster analysis of different tumors and normal tissues, we have found that NB and PCC tissues are more similar to each other than to any other tumor entity. This observation is not surprising, as both NB and PCC are neural crest-derived tumors (Figure 1).

**Figure 2:**
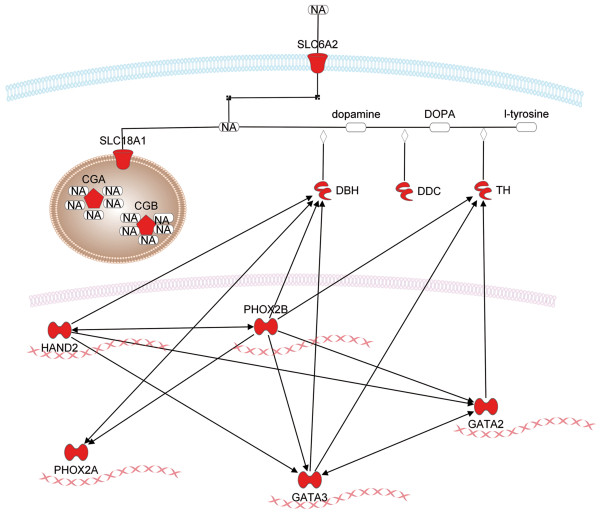
**Pathways with altered gene expression patterns involved in the regulation, synthesis, transport and storage of noradrenalin in NB and PCC.** Red labeled genes were overexpressed in more than 50 cases between these two entities and other tumors.

By the inspection of gene expression changes between NB, PCC and normal tissues and other tumors, the most characteristic gene expression feature of NB-PCC has been clearly related to the noradrenalin (NA) biosynthesis and neural crest cell development (Figure 2). NA is synthesized in both NB and PCC tissues, and several members of this synthetic pathway have been reported previously in NB as top scoring classifiers [9,33,34]. The significance of this pathway is well characterized in NB [10], however, the expression of several of its key members have not been reported in PCC, yet.

The homeobox transcription factor PHOX2B is essential for the differentiation and maintenance of noradrenergic neurons [53]. *PHOX2B* mutations are observed in rare hereditary forms of NB and Hirschsprung’s disease [54]. PHOX2B is involved in the transcriptional regulation of *RET* expression [55], however mutations of *PHOX2B* in hereditary and sporadic PCC have not been reported, yet.

The downstream targets of the master regulators of neural crest-derived precursor cell development PHOX2B and HAND2 are *PHOX2A*, *GATA2*, *GATA3*. These transcription factors play important roles in the expressional regulation of the *TH* and *DBH* genes [56]. TH catalyzes the rate limiting step of NA production, and DBH converts dopamine to NA. NA in chromaffin cells is co-stored in secretory granules with CGA and CGB molecules. The uptake into granules is mediated by SLC18A1 protein from the cytoplasm and by SLC6A2 from the extracellular space [10].

By the comparison of NB or PCC tissues with other tumors and normal tissues, we have found the significant overexpression of all the above mentioned genes in NB or PCC samples in more than 90 comparisons. This observation highlights the common origin of NB and PCC, however, it is surprising that there are only very few data on the expression of these genes in PCC.

### Similarities between NB and PCC

**Figure 3:**
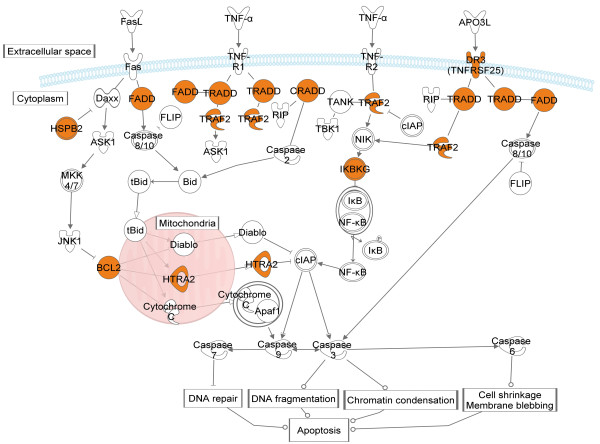
**Pathways with similar gene expression patterns involved in death receptor signaling among NB and PCC.** Yellow labeled genes expression were highly similar between these two entities.

To investigate the most significant similarities between NB and PCC tissues, we have searched for previously unreported reference genes. By this approach, the most similar pathway between NB and PCC was death receptor signaling (Figure 3).

Avoidance of apoptosis is a key process of tumorigenesis. Since this pathway included the most similar gene expression patterns in NB and PCC, we may conclude that these tumors may use the same strategies for rescue from programmed cell death.

The importance of proteins involved in the regulation of apoptosis triggered by death receptors (DRs) has been already reported in NB. Apoptosis in mammalian cells can be initiated through two major pathways: one involving tumor necrosis factor alpha (TNFα) and DR1-6, the other involving release of cytochrome c from mitochondria through Fas activation [57].

Numerous reports underline the significance of mitochondria-dependent signaling in NB. Fas resistance in NB may develop by the inactivation of caspase 8, which is absent in more than one third of NB cases [58], and often methylated in more than 60% of PCC [57]. Fas resistance may even develop in caspase 8-expressing NB via high level expression of the antiapoptotic protein BCL2 [58]. In the rat pheochromocytoma PC12 cell line, *BCL2* overexpression inhibits apoptosis [59]. We have found very similar expression levels of *BCL2* in the investigated NB and PCC tissues. Further members of mitochondria-dependent signaling were also similar between NB and PCC including Fas-associated via death domain (*FADD*), HtrA serine peptidase 2 (*HTRA2*), heat shock 27kDa protein 2 (*HSPB2*). These similarities raise the possible involvement of BCL2-mediated Fas resistance and mitochondria-dependent apoptotic pathway in PCCs pathogenesis, as well.

Underexpression of death receptor 3 (*TNFRSF15*) in NB has been reported previously, and correlated with the frequent deletion of chromosome region 1p36 [60] which chromosomal aberration is also known in sporadic and MEN2A-associated PCC [61]. The expression of DR3 has been very similar in NB and PCC in our analysis.

### Differences of NB and PCC

**Figure 4:**
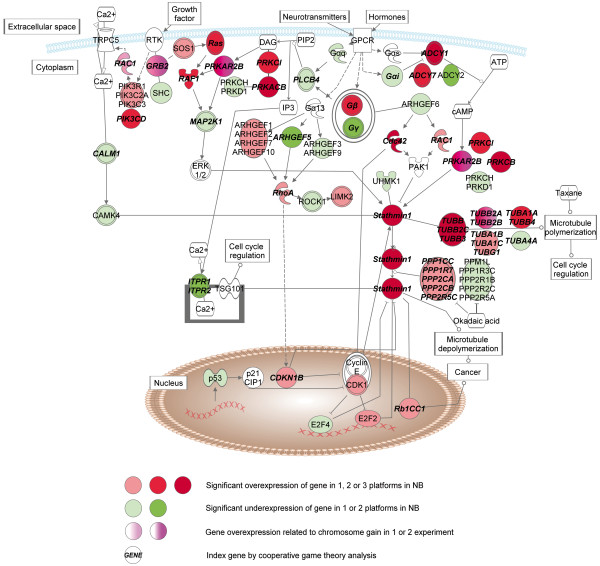
Pathways with altered gene expression patterns involved in Stathmin 1 signaling in NB compared to PCC.

Cancer regulation by Stathmin 1 (Figure 4) turned out to be the most significantly differentially affected pathway between NB and PCC. Stathmin 1 (*STMN1*) is overexpressed in a broad range of malignances [62]. Its expression is higher in benign PCC than normal adrenal medulla, and higher in malignant then benign PCC [63]. Stathmin 1 is involved in the regulation of microtubule dynamics by promoting depolymerization of microtubules and preventing polymerization of tubulin heterodimers. Both activities are regulated by mitogen-activated protein kinase (MAPK)-, calmodulin-dependent protein kinase (CAMK)-, p21 protein activated kinase 1 (PAK1)- and protein kinase A (PRKA)-mediated phosphorylation, which inactivate stathmin 1, and thereby prevent its binding to tubulin and interfere with the sensitivity of cancer cells to anti-microtubule drugs [64].

Phosphorylation of stathmin 1 at the beginning of mitosis is pivotal for allowing microtubules to polymerize and assemble into a mitotic spindle, whereas its dephosphorylation by protein phosphatases (PPIs) is critical for cells to exit mitosis. A similar effect is also shown by taxanes and vinca alkaloids, used for the chemotherapy of several cancers including NB [65].

By the comparison of NB and PCC, we have observed mainly the overexpression of genes involved in stathmin phosphorylation and dephosphorylation, tubulins and stathmin 1 in NB. These changes may highlight important differences in cell cycle regulation and chemoresistance between benign PCC and malignant NB via the enhanced regulation of stathmin function.

### Differences between early, late stage, MYCN-amplifying, non-amplifying NB

**Figure 5:**
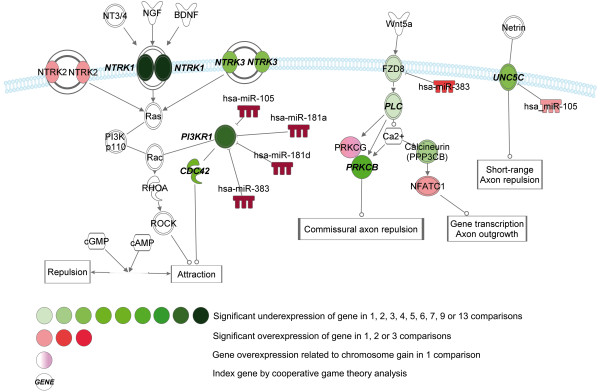
**Pathways with altered gene expression patterns of neurotrophic tyrosine kinase receptor (*NTRK*) frizzled family receptor 8 (*FZD8*) and unc-5 homolog C (*UNC5C*) genes involved in Axonal guidance signaling in late stage,*MYCN*-amplifying and unfavorable NB.**

**Figure 6:**
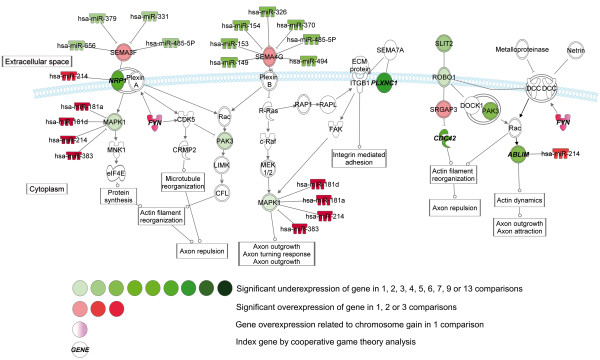
**Pathways with altered gene expression patterns of neuropilin 1 (*NRP1*), plexin C1 (*PLXNC1*) and roundabout, axon guidance receptor (*ROBO1*) genes involved in Axonal guidance signaling in late stage,*MYCN*-amplifying and unfavorable NB.**

**Figure 7:**
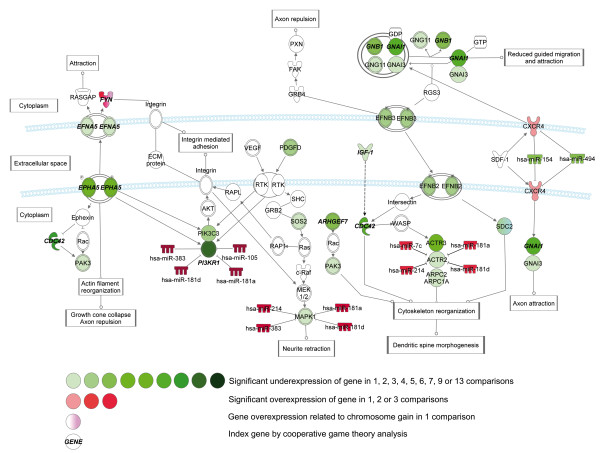
**Pathways with altered gene expression patterns of ephrin (*EFNA5, EFNB2, EFNB3*) and chemokine receptor 4 (CXCR4) genes involved in Axonal guidance signaling in late stage,*MYCN*-amplifying and unfavorable NB.**

Within the NB group, we have focused on the comparison of early and late stages, *MYCN*-non-amplifying and amplifying, and unfavorable and favorable NB. By this approach, we have observed the underexpression of axonal guidance pathway members in late stage, *MYCN*-amplifying and unfavorable NB (Figures 5, 6, 7).

Neuronal connections are formed by migrating axons, and the growth edge contains receptors which help to navigate the axon to its final destination. Several attractive and repulsive guidance cues and receptors have been identified for example roundabout 1 (ROBO1), plexin, neurophilin, ephrin (EFN) and neurotrophic tyrosine kinase (NTRK) receptors [66].

Among the receptors involved in axonal guidance, the underexpression of *NTRK1* (TrkA) was the most common (observed in more than ten datasets) alteration in *MYCN*-amplifying and unfavorable NB (Figure 5). We have also observed the underexpression of *NTRK3* (TrkC) in three cases and the overexpression of *NTRK2* (TrkB) in one. High *NTRK1* and *NTRK3* and low *NTRK2* expression in NB was associated with favorable clinical features and inversely associated with *MYCN* amplification [67]. Among downstream members of NTRK signaling, we have also observed the underexpression of *PIK3R1* in eight, *PIK3C3* in two, *PLCB1* and *PLCD4* in 1–1 dataset in late stage, *MYCN*-amplifying and unfavorable NB. The underexpression of *PIK3R1* was correlated with the overexpression of microRNAs *hsa-miR-105*, *hsa-miR-181a*, *hsa-miR-181d* and *hsa-miR-383* in these cases and it was a very frequent expression change in our in silico analysis, however, its significance in NB pathogenesis has not been described yet.

Significant expressional alterations of other receptors involved in neuronal migration were also observed. Underexpression of plexin receptor C1 (*PLXNC1*), a receptor characterized as a tumor suppressor in melanoma [68] was observed in late stage and *MYCN*-amplifying NB in seven cases (Figure 6). The underexpression of *EFNB2* and *EFNB3* was observed in two, and the underexpression of ephrin receptor A5 (*EPHA5*) in five datasets between early and late stage, and *MYCN*-non-amplifying and amplifying NB, respectively (Figure 7).

Neurophilin-1 (NRP1) receptor is involved in neuronal development and angiogenesis; and it binds semphorin3 proteins and VEGF. Significant downregulation of *NRP1* in late stage and *MYCN*-amplifying NB was noted in five datasets, and downregulation of *NRP1* in these cases was correlated with the overexpression of *hsa-miR-214* (Figure 6).

In our in silico analysis, we have observed several novel gene expression changes. The expressional alterations of axonal guidance pathway may be important in the development of NB metastases via the disintegration of cell-cell connections and highlight some potential therapeutic targets in unfavorable cases.

### Differences between SDH/VHL-, MEN2/NF1-related PCC

**Figure 8:**
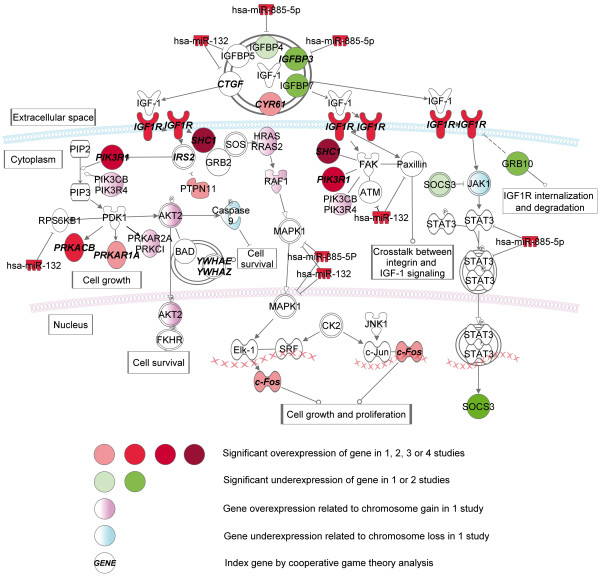
Pathways with altered gene expression patterns involved in IGF1 signaling in MEN2/NF1-related PCC compared to SDH/VHL-related ones.

By the comparison of SDH/VHL- and MEN2/NF1-related PCC, representing the two major pathogenic pathways of PCC, the overexpression of genes involved in IGF1 signaling in MEN2/NF1-related tumors has turned out as the most significant pathway (Figure 8). IGF1 promotes cell proliferation, growth and survival in several tissues. Seven binding proteins (IGFBP1-7) are involved in the control of its activity [69]. CYR61 (cysteine-rich, angiogenic inducer, 61), a secreted protein involved in the regulation of angiogenesis is also able to bind IGF1 with low affinity [70].

The IGF1 receptor (IGF1R) is a transmembrane tyrosine kinase protein. The activated IGF1R phosphorylates SHC, which binds to RET and growth factor receptor-bound protein 2 (GRB2), leading to activation of RAS-MAPK signaling. Previous studies confirmed that SHC binding to RET is crucial for the transforming activity of RET mutant proteins [71]. The enhanced IGF-1 signaling in RET-related PCC may be an important parallel pathway in the pathogenesis, which leads to the activation of RET via SHC. In MEN2/NF1-related PCC, downregulation of *IGFBP3* and *IGFBP7* was observed in two studies and the downregulation of *IGFBP4* and *IGFBP5* was correlated with the overexpression of *hsa-miR-132* and *has-miR-885-5p*. The overexpression of *IFG1R* in MEN2/NF1-related PCC was observed in two studies.

The overexpression of *SHC1* and *RET* was observed in all four comparisons in MEN2/NF1-related PCC. We have also observed the overexpression of *C-FOS* in these tumors as a possible outcome of the enhanced signaling activities of SHC/GRB/RAS complexes. Furthermore, the overexpression of *HRAS* and *RRAS2* was correlated with the frequent loss of chromosome region 11p15 in VHL PCC [72].

### Differences between MEN2A- and VHL -related PCC

**Figure 9:**
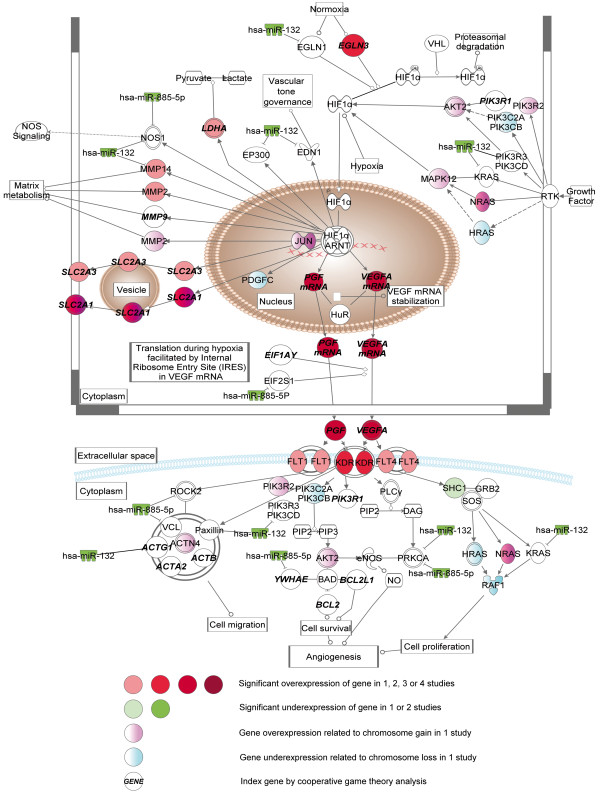
Pathways with altered gene expression patterns involved in VEGF and HIF1-α signaling in VHL-related PCC compared to MEN2A-related ones.

The comparison of MEN2A- and VHL- related PCC showed the significance of VEGF and HIF1-α signaling. As these pathways overlap at several points, we discuss them together (Figure 9).

HIF1-α is a transcription factor that transactivates genes participating in responses to hypoxia [73]. VHL is involved in the degradation of HIF1-α protein. Under normoxia, EGL nine homolog (EGLN) proteins are activated and hydroxylate HIF1-α, which allows VHL protein to bind and ubiquitinate HIF.

By the comparison of MEN2A- and VHL-related PCC, we have observed the significant overexpression of *EGLN3* in two studies and the overexpression of *EGLN1* was correlated with the downregulation of *hsa-miR-132* in VHL PCC. These gene expression changes may lead to enhanced hydroxylation of HIF1-α. The overexpression of HIF1-α target genes was also observed in VHL PCC as matrix metalloproteinase 2 (*MMP2*) and glucose transporter *GLUT1*. *GLUT1* overexpression in VHL could be correlated with the frequent loss of chromosome 1p34 often observed in MEN2A PCC [74]. Immunohistochemical analysis of *GLUT1,* however, failed to detect the protein in chromaffin cells [75]. The overexpression of *MMP2* was reported previously in several cancer tissues, and the inhibition of MMP2 function by halofuginone resulted in significant reduction of vascular functionality, decreased vascular density and less tumor size in VHL PCC *in vivo* models [76].

The overexpression of two VEGF genes, *VEGFA* and placental growth factor (*PGF*) were observed in three independent studies in VHL-related PCC. Overexpression of *PGF* was observed in several cancers. Reduced vascularisation and size of tumors were observed in *PGF* deficient mice [77]; however, its effect on tumor growth and angiogenesis is controversial. Binding of PGF to VEGFR1 leads to crosstalk between VEGFR1 and VEGFR2 and enhances VEGF-driven VEGFR2 signaling. PGF forms heterodimers with VEGF that do not display significant angiogenic effects, reducing the formation of VEGF-VEGF homodimers and lacks the ability of to induce angiogenesis by VEGFR2 activation [78].

The significance of HIF1-α and VEGF signaling is well characterized in VHL PCC, and our results may support and supplement these previous findings. Furthermore, these data corroborate the significance and authenticity of our in silico analysis results.

## Conclusions

The integration of different data sources from the same entities is one of the greatest challenges in contemporary molecular biology. We have applied several bioinformatics approaches to compare and integrate the genetic and transcriptional data from NB and PCC. We have demonstrated the usefulness of reference gene analysis for the identification of similarities among different entities and cooperative game theory analysis as a good method for the supplementation of results by conventional statistical analysis. By the application of these techniques we have performed a complex, integrative analysis which revealed several new pathogenic pathways. Our study has revealed several potential novel genes and pathways that might have major roles in NB and PCC pathogenesis and progression. The role of Stathmin 1 signaling in the pathogenesis of NB and PCC requires experimental validation to characterize its importance in these tumors. The relevance of *PHOX2B* gene and protein has not been studied in the pathogenesis of PCC yet, despite having fundamental role in the neural crest-derived precursor cell development. IGF1 signaling in MEN2/NF1-related PCC would also be an interesting topic to investigate. These pathways might even include potential novel therapeutic targets.

## Competing interests

The authors declare no conflict of interest.

## Authors' contributions

PMS and PI designed research. PMS, MP, DRS, AZ, AP performed data analysis. PMS, AF, KR and PI wrote the paper. All authors read and approved the final manuscript.

## Pre-publication history

The pre-publication history for this paper can be accessed here:

http://www.biomedcentral.com/1755-8794/5/48/prepub

## Supplementary Material

Additional file 1 **Significantly enriched pathways of the Ingenuity Pathway Analysis in different comparisons.** Ratio is calculated as follows: number of significanty differentially expressed genes in a given pathway, divided by total number of genes that make up that pathway. Click here for file

Additional file 2 **Significant gene expression changes between NB or PCC and other normal tissues and tumors (positive fold change values are marking the up-, negatives the downregulated genes in NB or PCC).** Genes are ranked by the number of significant expression changes between NB or PCC and other normal tissues and tumors. Click here for file

Additional file 3 **Similar gene expressions among NB and PCC.** Genes are ranked by their incidence in the different studies. Click here for file

Additional file 4 **Common gene expression changes between NB and PCC (positive fold change values mark the up-, negatives the downregulated genes in PCC).** Genes are ranked by the incidence of significant expression changes among the different studies. Click here for file

Additional file 5 **Common gene expression changes between different subgroups of NB (positive fold change values mark the up-, negatives the downregulated genes in late stage, MYCN-amplified or unfavorable NB).** Genes are ranked by the incidence of significant expression changes among the different studies. Click here for file

Additional file 6 **Common gene expression changes between SDH/VHL- and RET/NF1-related PCC (positive fold change values mark the up-, negatives the downregulated genes in RET/NF1-related PCC).** Genes are ranked by the incidence of significant expression changes among the different studies. Click here for file

Additional file 7 **Common gene expression changes between MEN2A- and VHL-related PCC (positive fold change values mark the up-, negatives the downregulated genes in VHL-related PCC).** Genes are ranked by the incidence of significant expression changes among the different studies. Click here for file
